# Maxillary and mandibular odontogenic myxomas: case report

**DOI:** 10.11604/pamj.2022.42.103.34690

**Published:** 2022-06-08

**Authors:** Jinane Kharbouch, Zakaria Aziz, Zahira Benzenzoum, Mohamed Salah Koussay Hattab, Salma Aboulouidad, Saad Fawzi, Nadia Mansouri Hattab

**Affiliations:** 1 Maxillo Facial Surgery Department, University Hospital Center Mohammed VI, Marrakech, Morocco

**Keywords:** Myxoma, odontogenic tumor, surgery, case report

## Abstract

Maxillary myxomas are rare benign odontogenic tumors with a high potential for infiltration and destruction. Clinical and radiological manifestations are variable and non-specific and often lead to confusion with other benign and malignant lesions. We present here two cases of odontogenic myxoma of different localization (maxilla and mandible). In both cases, the patient presented with a progressively enlarging facial swelling without any neurologic disturbance or lymph nodes. On computed tomography, the lesions appeared as a large unilocular, homogeneous image causing a cortical thinning and rupture along with repression of surrounding tissues. The clinical and radiological features were poorly suggestive of precise nature, and only histological examination of biopsy specimen was able to reveal the diagnosis of myxoma. Given the large size of the tumors and the locally aggressive pattern, we have opted for large maxillecetomy and marginal mandibulectomy. In conclusion, myxoma is a slow growing but locally aggressive tumor that the clinician should keep in mind. As there is a lack of consensus regarding their treatment, we can suggest a conservative treatment for small non-extensive lesions, which consists of enucleation and curettage of the tumor; and a radical excision in case of large tumors given the high risk of recurrence.

## Introduction

Maxillary myxomas are rare benign odontogenic tumors of mesenchymal origin with a high potential for infiltration and destruction [1]. Their Clinical and radiological manifestations are variable and non-specific and often lead to confusion with other benign and malignant lesions. A range of clinical and radiological arguments, supported by histological examination, is necessary for an accurate diagnosis and an appropriate treatment strategy [2]. The aim of our work is to present two cases of odontogenic myxoma of different localization (maxilla and mandible) bringing together the specific clinical, radiological and histological elements likely to differentiate this lesion from other severe pathological entities.

## Patient and observation

### Patient 1

**Patient information:** a 25-years-old patient, with no particular pathological history, consulted for right cheek swelling that had been evolving for 1 year, without ocular or neurological signs or nasal obstruction.

**Figure 1:**
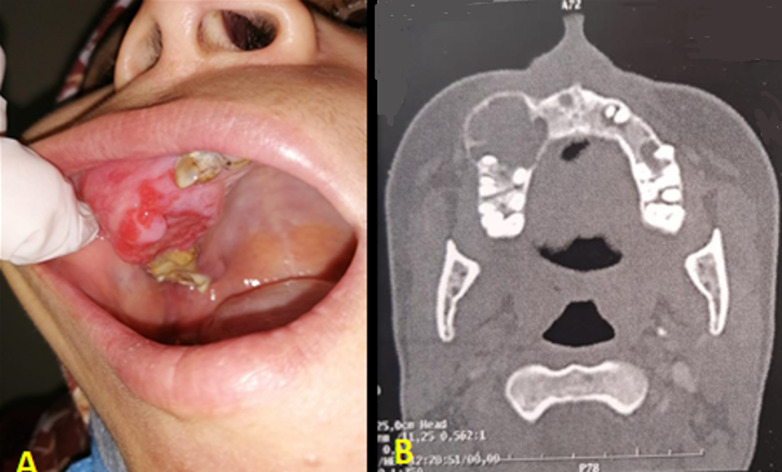
endobuccal view showing swelling of the upper right molar region occupying the corresponding vestibule area with inflammatory mucosa (A); computed tomography on axial view showing an osteolytic image of the right maxilla crossed by septas and causing thinning of the cortical (B)

**Clinical findings:** clinical examination showed facial asymmetry, due to non-painful swelling of the right cheek, fixed to underlying structures, with healthy covering skin. The intrabuccal examination showed a gingival mass located in the upper left premolar-molar region measuring 4 cm on the long axis, occupying the corresponding vestibule area and extending to the palatal side (Figure 1 A). The lesion was firm and painless. Poor oral hygiene with inflammatory mucosa was noticed. There was no sensory disturbance in the territory of the left infra-orbital nerve. Examination of the cervical lymph nodes showed no adenopathy.

**Diagnostic assessment:** the facial computed tomography revealed a unilocular osteolytic image of the upper maxilla, of a tissue density crossed by elongated and thin septa. The mass caused a cortical thinning of maxillary sinus walls with rupture and pushed back the surrounding soft tissues (Figure 1 B). In front of those non-specific features, an intraoral incisional biopsy was indicated and concluded to be an odontogenic myxoma.

**Therapeutic intervention:** given the large tumoral size, a right maxillectomy was performed under general anesthesia via a para-latero-nasal approach. Histological examination of the surgical specimen revealed a mesenchymal proliferation made up of stellate cells arranged on a myxoid background.

**Follow-up:** the postoperative course was without complications.

**Informed consent:** it was obtained from the patient.

### Patient 2

**Patient information:** a 35-years-old patient, with no particular pathological history, presented with progressively increasing swelling on the right side of the mandible.

**Figure 2:**
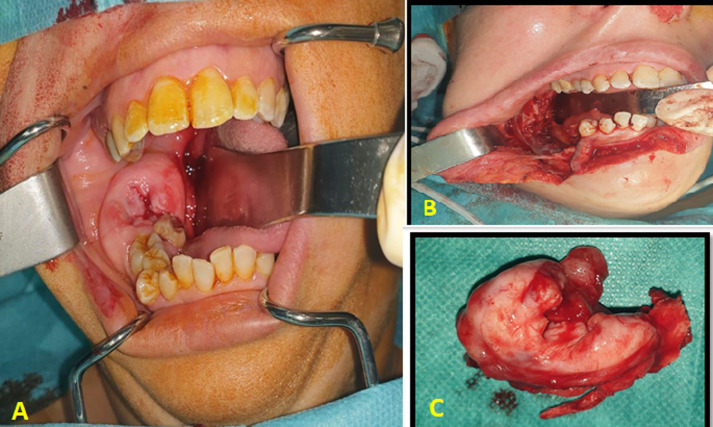
endobuccal preoperative view showing the swelling of the lower right molar sector with a displacement of the facing teeth (A); intraoperative view of the remaining cavity after tumoral resection (B); picture of the resection piece (C)

**Clinical findings:** clinical examination showed slight facial asymmetry. The intraoral examination revealed swelling extending from tooth 46 to the ramus covered by healthy-looking skin. The mass occupied the corresponding vestibule and caused lingual displacement. The covering mucosa looked normal (Figure 2 A). Upon palpation, the mass was firm and painless, with no dental mobility, and no sensory disturbances in the territory of the inferior alveolar nerve.

**Diagnostic assessment:** the panoramic X-ray discovered a relatively well-defined bilocular radiolucent image, directly located above tooth 46 and extending to the ramus. On facial computed tomography, the lesion appeared as a homogeneous bone formation with regular contours of the right half mandible, involving the horizontal branch and the lower 2/3 of the ramus. The mass caused a thinning and rupture of both inner and outer cortical.

**Therapeutic intervention:** given the aggressive and locally destructive aspect of the tumor, a marginal mandibulectomy was performed using a trans-labial approach (Figure 2 B, C). The anatomo-pathological study confirmed the diagnosis of an odontogenic myxoma.

**Follow-up:** two months after, the patient presented with a pathological fracture of the right angle fixed by a manipulation. As the follow-up showed no complications, a bony graft from the iliac crest was performed six months later.

**Informed consent:** it was obtained from the patient.

## Discussion

Myxoma is a benign, rare and locally invasive mesenchymal tumor, composed of stellate and spindle-shaped cells sitting in a myxoid stroma, with some collagen fibers; when the fibers are more abundant, it is called fibromyxoma [3]. It represents only 3 to 6% of benign odontogenic tumors and only 0.41% of bone tumors [4]. This lesion more frequently involves the mandible; in the angular and molar region; than the maxilla and, exceptionally, the soft parts (soft palate, lower lip, etc.) [2]. It generally affects adolescents and young adults, rarely children and exceptionally the elderly [5]. The age of our two patients reported in our study is 25 and 35 years old. The majority of authors report a slight female predominance which corresponds to our data Clinically, the myxoma is slow-growing, most often asymptomatic, thus delaying the consultation period, which according to the literature varies from one month to eight years [6]. In our observations, this period varied from 5 months to 1 year. It is locally manifested by a unilateral painless swelling without functional disorders, as in the case of our two patients.

Radiologically, it results in a clear, unilocular or multilocular radio image, with a “soap bubble” appearance. The bony septa located inside the lesion are thin and elongated. The cortical bone is thinned and deformed. In advanced lesions, this cortex is ruptured, leading to invasion of the adjacent soft tissues. The lesion pushes back the teeth, whose roots are rarely eroded. A computed tomography examination is essential to determine the modalities of the surgical intervention according to the three-dimensional topography of the lesion and its connexion to adjacent structures. On magnetic resonance imaging, myxomas have a variable, often heterogeneous signal. A hyposignal in T1 and hypersignal T2 is very evocative but inconstant [7,8]. Macroscopically, the myxoma is grayish, gelatinous in consistency. On histological study, it is composed of triangular or stellate connective cells, anastomosed by fine extensions and embedded in abundant mucoid material [8].

The diagnosis of myxoma is histological, the appearance of these tumors is characterized by the presence of round and angular cells; basophilic cytoplasm, scanty, with a small hyperchromatic nucleus; disseminated in an abundant myxoid stroma containing mucopolysaccharides and a network of reticular fibres [9]. For some authors, presence of an odontogenic epithelium within the tumor pleads for a dental origin. The differential diagnosis of myxoma arises essentially with ameloblastoma but also with sarcoma, chondroma, fibroma, giant cell tumors and intraosseous hemangioma. The treatment of choice for myxoma is surgical excision. The conservative treatment is mainly indicated in small non-extensive lesions; which consists of careful enucleation and curettage, with or without chemical or electrical cauterization of the walls of the post-resection remaining cavity. However, this treatment is accompanied by a higher risk of recurrence, estimated at 25% according to the various studies [10]. For this reason, some authors consider radical treatment with security margins of 1.5 cm from the limits of the lesion, but it results in a loss of bone substance requiring reconstructive surgery. Reossification could be observed radiologically from the 6th postoperative month.

Recurrence is more frequently observed in the two years following the surgical treatment; hence the importance of regular and prolonged postoperative clinical and radiological monitoring. A six-monthly rhythm for five years is advocated.

## Conclusion

The diagnosis of myxoma can be suggested based on clinical and radiological data, but the confirmation is histological. Although this lesion is rare, it should not be overlooked. It´s a slow-growing but locally aggressive tumor since it is a poorly circumscribed non-encapsulated tumor. Its treatment is exclusively surgical; we can suggest a conservative treatment for small non-extensive lesions, which consists of enucleation and curettage of the tumor; and a radical excision in case of large tumors.

## References

[1] Wong GB (1992). Large odontogenic myxoma of the mandible treated by sagittal ramus osteotomy and peripheral ostectomy. J Oral Max fac Surg.

[2] Bayi EH, El Harti K, Chbicheb S, El Wady W (2006). Myxome odontogène des maxillaires: Odontogenic myxoma of the maxillary. Revue de Stomatologie et de Chirurgie Maxillo-faciale.

[3] Buchner A, Odell EW, Barnes L, Eveson J, Reichart P, Sidransky D (2005). Odontogenic myxoma-Myxofibroma. Head and Neck Tumors.

[4] Sentilhes CI, Michaud J (1987). Myxomes des maxillaires. Rev Stomatol Chir Maxillo fac.

[5] Farman (1977). Myxofibroma of the jaws. Br J Oral Surg.

[6] Schneck DL, Gross PD, Tabor MW (1993). Odontogenic myxoma: report of two cases with reconstruction considerations. J Oral Maxillo fac Surg.

[7] Kim JD, Kim KW, Lim SH (2004). Odontogenic myxoma: a case report with recent image modalities. Korean J Oral Maxillo fac Radiol.

[8] Asaumi J, Konouchi H, Hisatomi M, Kishi K (2001). Odontogenic myxoma of maxillary sinus: CT and MR-pathologic correlation. European Journal of Radiology.

[9] Rotenberga BW, Daniela SJ, Nishb IA, Nganc BY, Fortea V (2004). Myxomatous lesions of the maxilla in children: a case series and review of management. Int J of Pediatric Otorhinolaryngology.

[10] Lo Muzio L, Nocin P, Favia G, Procacemi M, Mignogna MD (1996). Odontogenic myxoma of the jaws: a clinical, radiologic, immunohistochemical and ultrastructural study. Oral Surg Oral Med Oral Pathol.

